# Metabolic syndrome and incident thyroid cancer risk in adults: A systematic review and meta-analysis

**DOI:** 10.17305/bb.2026.13977

**Published:** 2026-04-27

**Authors:** Zhengwen He, Jing Zhou, Xianxiang Lu

**Affiliations:** 1 Department of Endocrinology, The Fifth People’s Hospital of Zhangjiagang City, Zhangjiagang, China

**Keywords:** Thyroid cancer, metabolic syndrome, risk factor, insulin resistance, meta-analysis

## Abstract

The global incidence of thyroid cancer (TC) has increased, with metabolic syndrome (MetS) identified as a potential modifiable risk factor. However, epidemiological evidence regarding this association remains inconsistent. We conducted a meta-analysis to quantify the relationship between MetS and the risk of incident TC in adult populations. We searched PubMed, Embase, and Web of Science for longitudinal observational studies assessing MetS in relation to incident TC. Risk ratios (RRs) with 95% confidence intervals (CIs) were pooled using random-effects models to account for heterogeneity. Our analysis included six cohort studies comprising seven independent datasets and a total of 15,730,432 participants, among whom 83,351 incident TC cases were identified during follow-up periods ranging from 2.7 to 12.0 years. The results indicated that MetS was associated with a significantly increased risk of TC (RR = 1.26, 95% CI 1.10–1.46; *P <* 0.001), exhibiting substantial heterogeneity (I^2^ ═ 91%). This association was consistent across prospective and retrospective cohorts, varying sample sizes, follow-up durations, and different strata of TC incidence (all *P* for subgroup differences > 0.05). Moreover, sex-specific analyses revealed comparable associations in both men and women. Studies with higher methodological quality (Newcastle–Ottawa Scale score = 9) demonstrated a diminished yet still significant association compared to those with scores of 7–8 (RR = 1.15 vs. 1.80; *P* for subgroup difference < 0.001). In conclusion, MetS is associated with a moderately increased risk of TC in adults. However, due to the observational nature of the included studies and the potential for residual confounding, these findings should be interpreted as an association rather than definitive evidence of a causal relationship.

## Introduction

Thyroid cancer (TC) is the most prevalent malignancy of the endocrine system, with its incidence increasing significantly worldwide over the past several decades [1, 2]. Clinically, TC encompasses a diverse group of malignancies, including papillary, follicular, medullary, and anaplastic subtypes, which vary considerably in incidence, molecular characteristics, and clinical prognosis [3]. While enhanced diagnostic practices, such as the widespread use of high-resolution ultrasonography and fine-needle aspiration, have contributed to this increase, overdiagnosis alone cannot fully account for the observed trends [4]. Notably, TC generally has a favorable prognosis, especially for differentiated subtypes like papillary thyroid carcinoma, which exhibit high long-term survival rates [3]. However, the rising incidence presents a considerable public health burden due to the need for lifelong surveillance, treatment-related morbidity, and escalating healthcare costs [5, 6]. In this context, the identification of modifiable risk factors beyond established determinants—such as ionizing radiation exposure, female sex, iodine status, and genetic predisposition—has gained increasing significance [2]. Emerging evidence suggests that metabolic abnormalities may contribute to cancer development, prompting interest in their role in thyroid carcinogenesis [7].

Metabolic syndrome (MetS) is a clustered metabolic disorder characterized by central obesity, insulin resistance, dyslipidemia, and hypertension [8]. It is commonly diagnosed using standardized criteria, notably those proposed by the National Cholesterol Education Program Adult Treatment Panel III (NCEP-ATP III) and the International Diabetes Federation (IDF) [8, 9]. MetS has reached epidemic proportions globally, paralleling rising rates of obesity and type 2 diabetes [10]. Accumulating evidence indicates that MetS may promote carcinogenesis through various biological mechanisms, such as hyperinsulinemia, insulin-like growth factor signaling, chronic low-grade inflammation, oxidative stress, altered adipokine profiles, and hormonal dysregulation [11, 12], all of which could plausibly affect thyroid cell proliferation and tumor initiation. However, epidemiological evidence linking MetS as a composite exposure to TC risk remains limited and inconsistent [7]. A prior meta-analysis published in 2018 included only two studies specifically evaluating MetS and TC, reporting a null association primarily due to sparse data and heterogeneous exposure definitions [13]. Since then, several large population-based cohort studies with longitudinal follow-up have emerged, providing new and more robust evidence [14–17]. Consequently, the present meta-analysis aims to systematically synthesize updated longitudinal data to clarify the association between MetS and the risk of incident TC in adults, while also exploring potential sources of heterogeneity across study characteristics.

## Materials and methods

The meta-analysis was conducted in accordance with established methodological guidelines, adhering to the principles outlined in the Preferred Reporting Items for Systematic Reviews and Meta-Analyses (PRISMA) 2020 statement [18] and the Cochrane Handbook for Systematic Reviews and Meta-Analyses [19]. This encompassed protocol planning, study selection, data extraction, statistical analysis, and reporting. The study protocol was prospectively registered in the International Prospective Register of Systematic Reviews (PROSPERO) database (registration number: CRD420261298855).

### Database search

We performed a systematic and comprehensive literature search in PubMed, Embase, and Web of Science to identify studies relevant to this review. The search strategy was developed using three core concept domains: metabolic syndrome (including insulin resistance syndrome and syndrome X), thyroid, and malignant outcomes (including cancer, neoplasm, carcinoma, malignancy, tumor, and malignant). Eligibility was restricted to full-text, peer-reviewed studies published in English and conducted in human populations. Additionally, the reference lists of pertinent reviews and original articles were manually screened to identify further eligible studies. All databases were searched from their inception until 31 December 2025. The detailed search strategy for each database is provided in Supplemental file 1.

### Study inclusion and exclusion criteria

The selection of studies was guided by the PICOS framework:

- P (Population): Adult participants (≥ 18 years) from the general population without TC at baseline.
- I (Intervention/Exposure): MetS defined according to recognized diagnostic criteria, including but not limited to the NCEP-ATP III, IDF, or harmonized criteria.
- C (Comparison): Individuals without MetS at baseline.
- O (Outcome): Incident TC confirmed by medical records, cancer registries, or pathological diagnosis.
- S (Study design): Observational studies with longitudinal follow-up, including cohort studies, nested case-control designs, or post-hoc analyses of trials.

Studies were excluded if they met any of the following criteria: (1) cross-sectional studies, case series, case reports, editorials, reviews, conference abstracts, or animal studies; (2) studies evaluating individual components of MetS only (e.g., obesity, diabetes, hypertension) without defining MetS as a composite exposure; (3) studies lacking a non-MetS comparison group; (4) studies not reporting TC–specific outcomes; and (5) duplicate publications or overlapping populations, in which case the study with the largest sample size was included.

### Study quality evaluation and data extraction

Two investigators independently conducted the literature search, study screening, quality assessment, and data extraction, with any discrepancies resolved through discussion and consultation with the corresponding author. Study quality was assessed using the Newcastle–Ottawa Scale (NOS) [20], which evaluates selection, comparability, and outcome ascertainment; NOS scores range from 1–9, with studies scoring ≥ 7 considered high quality. Extracted data included study characteristics (first author, publication year, country, and study design), participant characteristics (population source, sample size, age, and sex), exposure assessment (diagnostic criteria for MetS), follow-up duration, outcome validation (methods for TC diagnosis and number of incident TC cases), incidence of TC expressed per 100,000 person-years where available, and covariates included in the adjusted analyses examining the association between MetS and TC risk.

**Figure 1.**
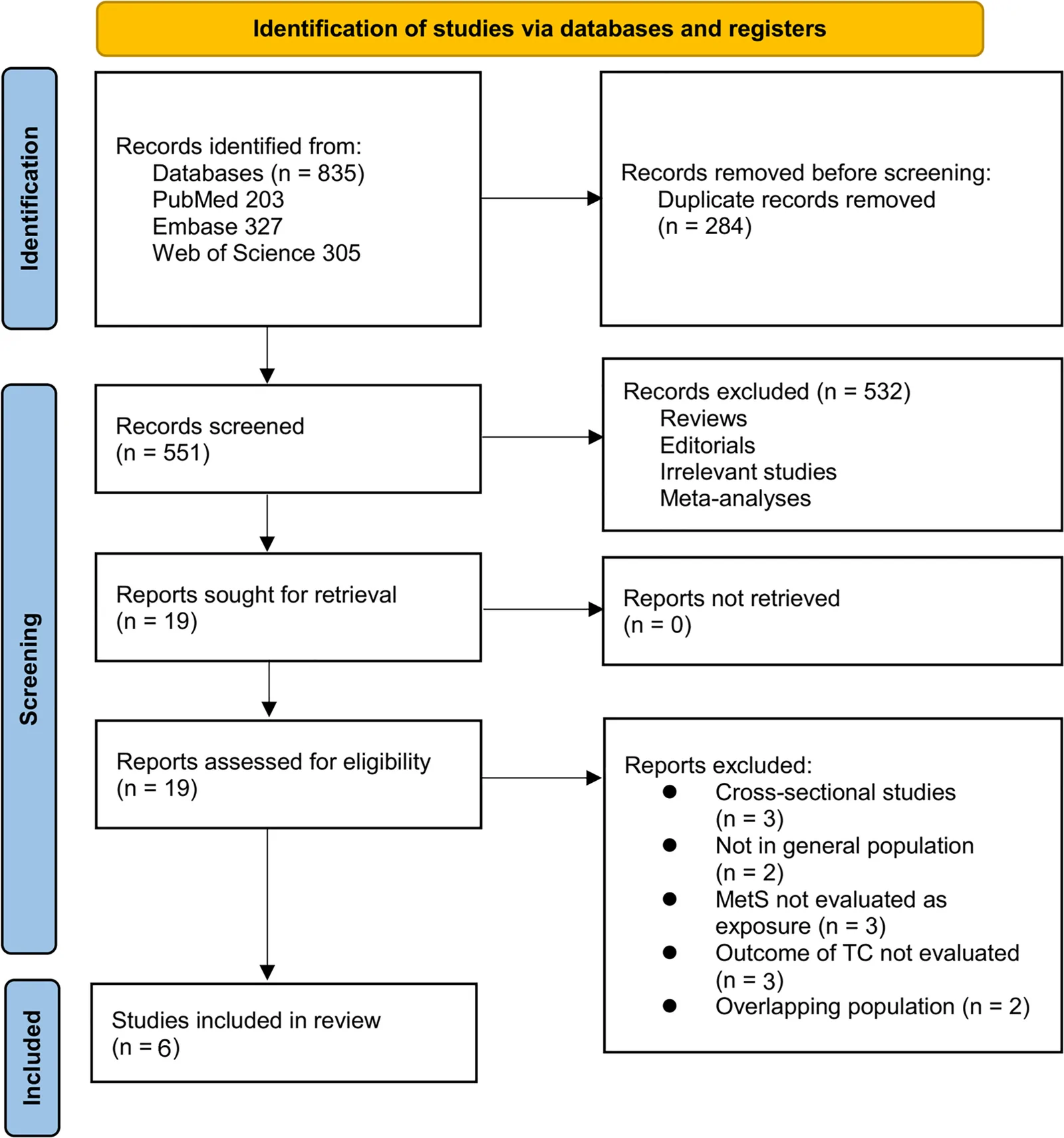
**Flow diagram illustrating the study selection process**.

### Statistical analyses

We assessed the association between MetS and the risk of TC in adult populations by pooling risk ratios (RRs) with corresponding 95% confidence intervals (CIs), comparing participants with MetS to those without MetS at baseline. For studies reporting hazard ratios (HRs), these were treated as equivalent to RRs and were directly pooled, as is conventional in meta-analyses of longitudinal studies with relatively low event rates [21]. When multiple models were reported, the estimates from the most fully adjusted model were extracted to minimize residual confounding. Effect estimates and their standard errors were calculated from reported 95% CIs or *P*-values and log-transformed prior to synthesis to improve normality and variance stability [19]. Statistical heterogeneity across studies was evaluated using the Cochrane *Q* test and quantified with the I^2^ statistic [22], with values <25%, 25%–75%, and >75% indicating low, moderate, and high heterogeneity, respectively. Summary estimates were generated using a random-effects model based on the inverse-variance method with the DerSimonian–Laird estimator to account for between-study variability [19]. Additionally, 95% prediction intervals (PIs) were calculated to estimate the range within which the true effect size of a future study is expected to lie, considering between-study heterogeneity [19]. Sensitivity analyses were performed by sequential exclusion of individual studies to examine the robustness of the results [19]. Prespecified subgroup analyses explored potential sources of heterogeneity based on study design (prospective vs. retrospective), sample size, participant sex, mean follow-up duration, overall TC incidence, and NOS score. For continuous study-level variables, subgroup cutoffs were informed by the distribution of values across included studies, with approximate median values used as a reference. To enhance interpretability and ensure meaningful group comparisons, these thresholds were defined using pragmatic, rounded cutoffs (e.g., sample size of 1,000,000, follow-up duration of 7 years, and incidence of 30 per 100,000 person-years), rather than exact median values. Publication bias was assessed through visual inspection of funnel plots and Egger’s regression test [23]. Statistical significance was defined as a two-sided *P* value < 0.05. All analyses were performed using Review Manager (RevMan) version 5.3 (Cochrane Collaboration, Oxford, UK) and Stata version 17.0 (StataCorp, College Station, TX, USA).

**Table 1:** Characteristics of the cohort studies included in the analysis

**Study**	**Country**	**Study design**	**Population source**	**Sample size**	**Mean age (years)**	**Men (%)**	**Diagnosis of MetS**	**Mean follow-up (years)**	**No. of patients with TC**	**Incidence of TC (per 100,000 person year)**	**Methods for TC validation**	**Variables adjusted**
Russo 2008	Italy	RC	Community population aged 40 years or older	16,677	NR	NR	NCEP-ATP III	2.7	7	15.55	Cancer Registry (ICD-10 coded)	Age and sex
Almquist 2011 men	Norway, Austria, Sweden	PC	Community-based population	289,866	43.9	100	NCEP-ATP III	12	133	3.82	National Cancer Registries (ICD-7 code 194)	Age, sex, and smoking
Almquist 2011 women	Norway, Austria, Sweden	PC	Community-based population	288,834	44.1	0	NCEP-ATP III	12	255	7.36	National Cancer Registries (ICD-7 code 194)	Age, sex, and smoking
Park 2020	South Korea	RC	Community-based health insurance database participants aged 20 years or older	9,890,917	NR	55.3	NCEP-ATP III	7.2	77,133	108.31	NHIS hospitalization records (ICD-10 C73)	Age, sex, smoking, alcohol, physical activity, income, and CKD
Nguyen 2022	South Korea	PC	Community population aged 40 years or older	160,650	NR	34.4	NCEP-ATP III	7.4	471	39.62	Clinically validated	Age, sex, smoking, alcohol, physical activity, education, menopausal status (women), hormone therapy
López-Jiménez 2024	Spain	RC	Primary care population	3,918,781	55.4	39.1	NCEP-ATP III	4	2707	17.27	Local Cancer Registries	Age, sex, smoking status, alcohol intake, and nationality
Han 2025	South Korea	RC	Community-based population aged 75 years or older	1,164,707	78.7	39.7	NCEP-ATP III	6.9	2645	32.91	National Cancer Registry (ICD-10 coded)	Age, sex, income, smoking status, alcohol consumption, physical activity, and CKD

The study conducted by Almquist et al. (2011) utilized a single prospective cohort and reported results separately for men and women, treating these as two independent datasets in the meta-analysis. **Abbreviations:** CKD, chronic kidney disease; ICD, International Classification of Diseases; MetS, metabolic syndrome; NCEP-ATP III, National Cholesterol Education Program Adult Treatment Panel III; NHIS, National Health Insurance Service; NR, not reported; PC, prospective cohort; RC, retrospective cohort; TC, thyroid cancer.

## Results

### Database search results

The study identification and selection process is illustrated in Figure 1. A total of 835 records were identified through searches across three databases, with 284 duplicates removed. Following title and abstract screening, 532 records were excluded for not meeting the predefined eligibility criteria. Subsequently, 19 articles were reviewed in full text by two independent reviewers, resulting in the exclusion of 13 studies for reasons detailed in Figure 1. Ultimately, six studies were deemed eligible and included in the quantitative meta-analysis [14–17, 24, 25].

### Summary of study characteristics

The main characteristics of the included studies are summarized in Table 1. The meta-analysis comprised six cohort studies, consisting of two prospective cohorts [14, 15] and four retrospective cohorts [16, 17, 24, 25]. Notably, the prospective study by Almquist et al. (2011) reported outcomes separately for men and women; thus, these sex-specific estimates were treated as independent datasets and included separately in the analyses [14]. These studies were published between 2008 and 2025 and were conducted across Europe and East Asia, including Italy, Norway, Austria, Sweden, Spain, and South Korea. All studies were population-based, drawing participants from community cohorts [15, 17, 24, 25], national health insurance databases [14], or primary care registries [16]. Collectively, the studies included 15,730,432 participants, with sample sizes ranging from 16,677 to 9,890,917. Where reported, mean age varied significantly across studies, reflecting broad population coverage from middle-aged adults to elderly individuals aged ≥ 75 years, with the proportion of men ranging from 34.4% to 100%. MetS was consistently defined using the NCEP-ATP III criteria across all included studies, enhancing comparability. Mean follow-up durations ranged from 2.7–12.0 years. TC outcomes were ascertained using validated methods, including national or local cancer registries [16, 17, 24, 25], clinically validated diagnoses [15], or health insurance hospitalization records based on International Classification of Diseases (ICD) coding systems [14]. During follow-up, a total of 83,351 incident TC cases were reported, with incidence rates ranging from 3.82–108.31 per 100,000 person-years. All studies adjusted for key confounders, most commonly age and sex, with several studies [14–17, 25] further accounting for lifestyle factors, socioeconomic indicators, and comorbidities such as smoking, alcohol consumption, physical activity, income, menopausal status, hormone therapy, and chronic kidney disease (CKD).

### Study quality evaluation

Study quality was assessed using the NOS, with results presented in Table 2. NOS scores ranged from 7 to 9, indicating overall good methodological quality across the included studies. All studies received full points for representativeness of the exposed cohort, ascertainment of MetS, confirmation that TC was not present at baseline, and outcome assessment. Four studies [14, 15, 17, 25] achieved high NOS scores of 9, reflecting robust cohort selection, comprehensive adjustment for major confounders, reliable outcome ascertainment, and sufficient follow-up duration. One study [24] scored 7, primarily due to limited control for additional confounding factors and shorter follow-up. Another study scored 8 [16] due to inadequate follow-up duration. Overall, all included studies were considered to be of high or acceptable quality, supporting the reliability of the pooled estimates examining the association between MetS and the risk of TC.

**Table 2:** Evaluation of study quality using the Newcastle–Ottawa scale

**Study**	**Representativeness of the exposed cohort**	**Selection of the non-exposed cohort**	**Ascertainment of exposure**	**Outcome not present at baseline**	**Control for age and sex**	**Control for other confounding factors**	**Assessment of outcome**	**Enough long follow-up duration**	**Adequacy of follow-up of cohorts**	**Total**
Russo 2008	1	1	1	1	1	0	1	0	1	7
Almquist 2011 men	1	1	1	1	1	1	1	1	1	9
Almquist 2011 women	1	1	1	1	1	1	1	1	1	9
Park 2020	1	1	1	1	1	1	1	1	1	9
Nguyen 2022	1	1	1	1	1	1	1	1	1	9
López-Jiménez 2024	1	1	1	1	1	1	1	0	1	8
Han 2025	1	1	1	1	1	1	1	1	1	9

### Meta-analysis results

The pooled results of the meta-analysis across seven datasets from six cohort studies [14–17, 24, 25] demonstrated that MetS in adult populations was associated with a significantly increased risk of TC (RR: 1.26, 95% CI: 1.10–1.46; *P <* 0.001; I^2^ ═ 91%; Figure 2). The 95% prediction interval was 1.02–1.56, suggesting that the true effect size may vary across populations, although the interval remained above unity. Given the substantial between-study heterogeneity, this estimate should be interpreted with caution. Sequential exclusion of individual studies did not materially alter the findings, with pooled RRs ranging from 1.15–1.33 (all *P <* 0.05). Further subgroup analyses revealed similar results between prospective and retrospective cohorts (RR: 1.17 vs. 1.34; *P* for subgroup difference = 0.37; Figure 3A), in studies with sample sizes greater than 1,000,000 and those with sizes less than or equal to 1,000,000 (RR: 1.34 vs. 1.17; *P* for subgroup difference = 0.36; Figure 3B), between men and women (RR: 1.17 vs. 1.10; *P* for subgroup difference = 0.44; Figure 4A), in studies with mean follow-up durations of less than 7 years and those of 7 years or more (RR: 1.44 vs. 1.14; *P* for subgroup difference = 0.24; Figure 4B), and in cohorts with overall TC incidence of less than 30 or 30 or more per 100,000 person-years (RR: 1.28 vs. 1.18; *P* for subgroup difference = 0.66; Figure 5A). Finally, the association appeared attenuated in studies with higher NOS scores (NOS of 9 vs. NOS of 7 or 8), although it remained statistically significant (RR: 1.15 vs. 1.80; *P* for subgroup difference < 0.001; Figure 5B).

**Figure 2.**
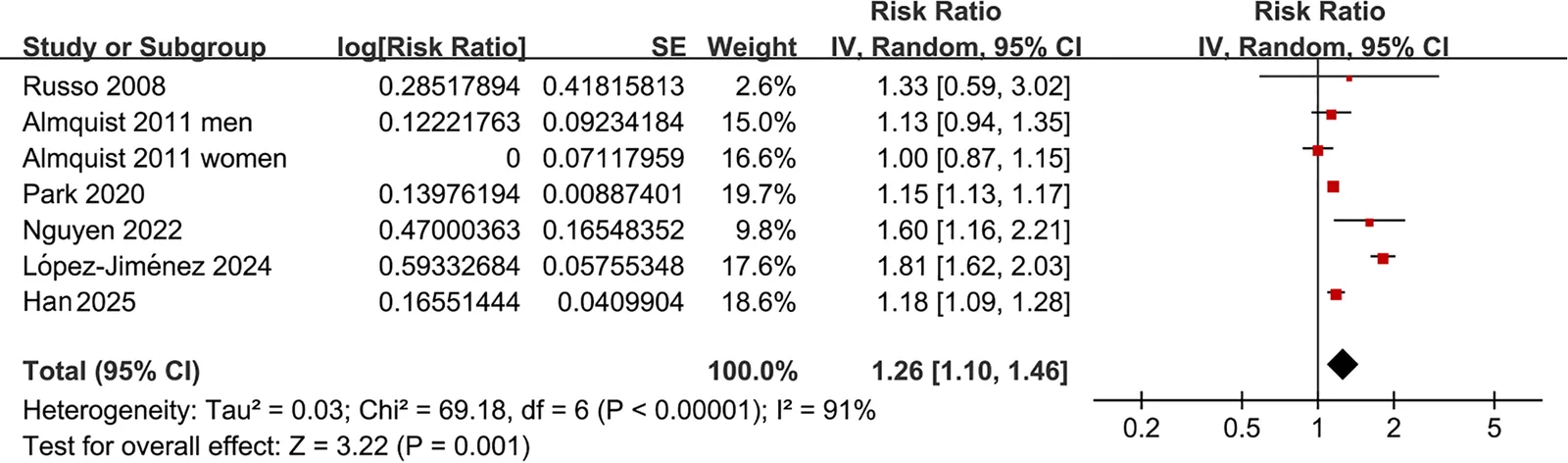
**Association between MetS and incident TC risk in adults.** Forest plot of seven datasets derived from six cohort studies evaluating the association between MetS and the risk of incident thyroid cancer in adult populations. The pooled analysis using a random-effects model showed that MetS was associated with a significantly increased risk of TC (RR 1.26, 95% CI 1.10–1.46), with substantial between-study heterogeneity (I^2^ ═ 91%). Squares indicate study-specific effect estimates, with square size proportional to study weight, horizontal lines represent 95% confidence intervals, and the diamond denotes the pooled effect estimate. Sex-specific estimates from Almquist et al. were analyzed separately for men and women. **Abbreviations:** CI, confidence interval; I^2^, inconsistency index; MetS, metabolic syndrome; RR, risk ratio; TC, thyroid cancer.

**Figure 3.**
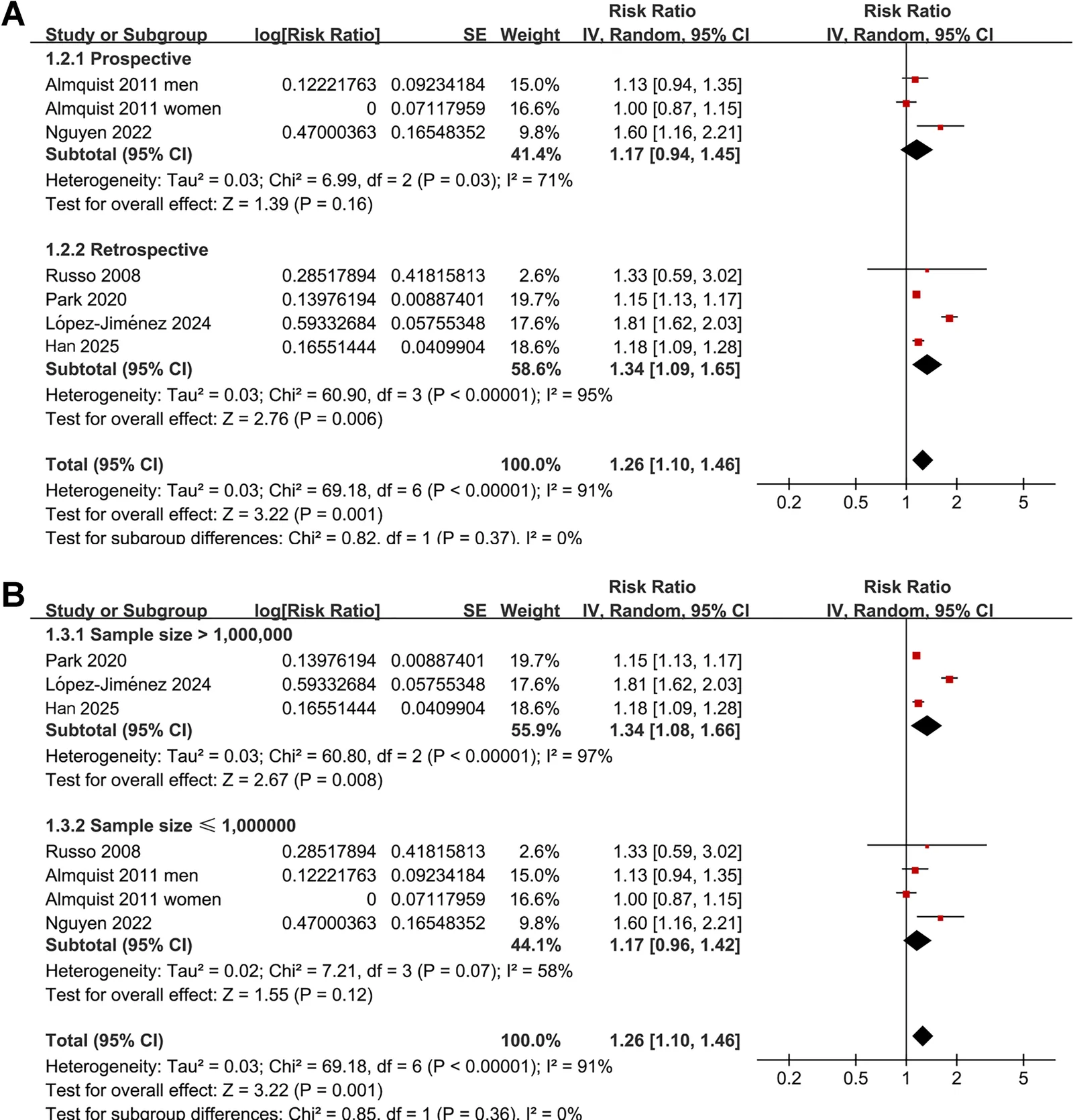
**Forest plots for subgroup analyses of the association between MetS and the risk of TC according to study design and sample size.** (A) Subgroup analysis stratified by study design, comparing prospective and retrospective cohorts. (B) Subgroup analysis stratified by sample size, comparing studies with >1,000,000 participants versus those with ≤1,000,000 participants. Across both stratifications, the direction of the association remained consistent, and no significant subgroup differences were observed. The study by Almquist et al. contributed two independent sex-specific datasets (men and women), which were analyzed separately. Squares represent study-specific effect estimates, with sizes proportional to study weight; horizontal lines indicate 95% CIs; and diamonds denote pooled subgroup and overall estimates derived from random-effects models. **Abbreviations:** CI, confidence interval; MetS, metabolic syndrome; RR, risk ratio; TC, thyroid cancer.

**Figure 4.**
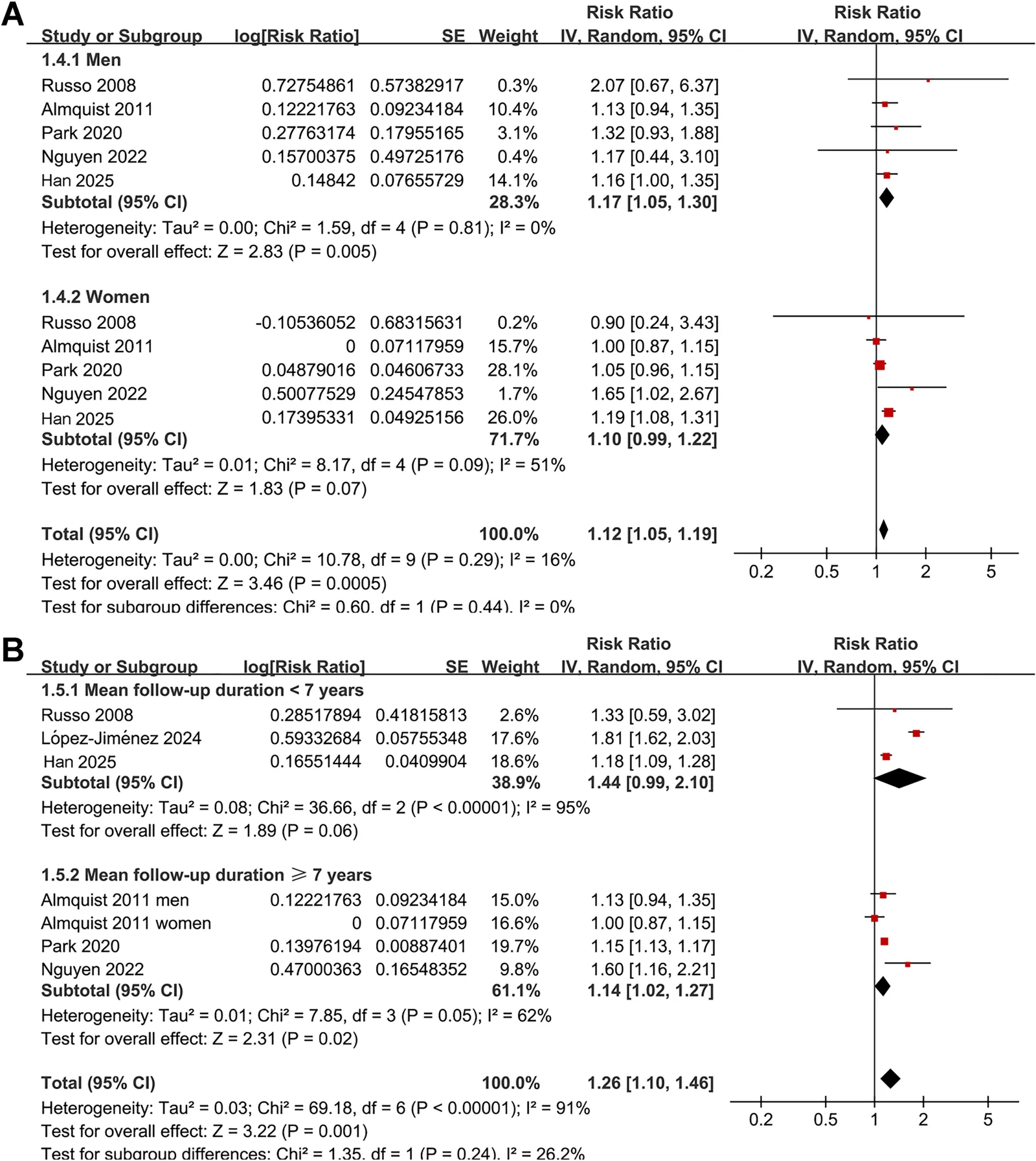
**Forest plots for subgroup analyses of the association between MetS and the risk of TC according to sex and mean follow-up duration.** (A) Sex-stratified subgroup analysis comparing men and women. The direction of the association was generally consistent across sexes, and no significant subgroup difference was observed. The “Total” estimate in panel A represents the pooled effect derived from sex-specific datasets in which men and women were analyzed separately, rather than the primary overall meta-analysis of all included datasets. (B) Subgroup analysis according to mean follow-up duration, comparing studies with a mean follow-up of < 7 years versus ≥ 7 years. The association between MetS and TC remained directionally similar across follow-up strata, with no significant subgroup difference. Squares represent study-specific effect estimates, with sizes proportional to study weight; horizontal lines indicate 95% CIs; and diamonds denote pooled subgroup and overall estimates generated using random-effects models. **Abbreviations:** CI, confidence interval; MetS, metabolic syndrome; RR, risk ratio; TC, thyroid cancer.

**Figure 5.**
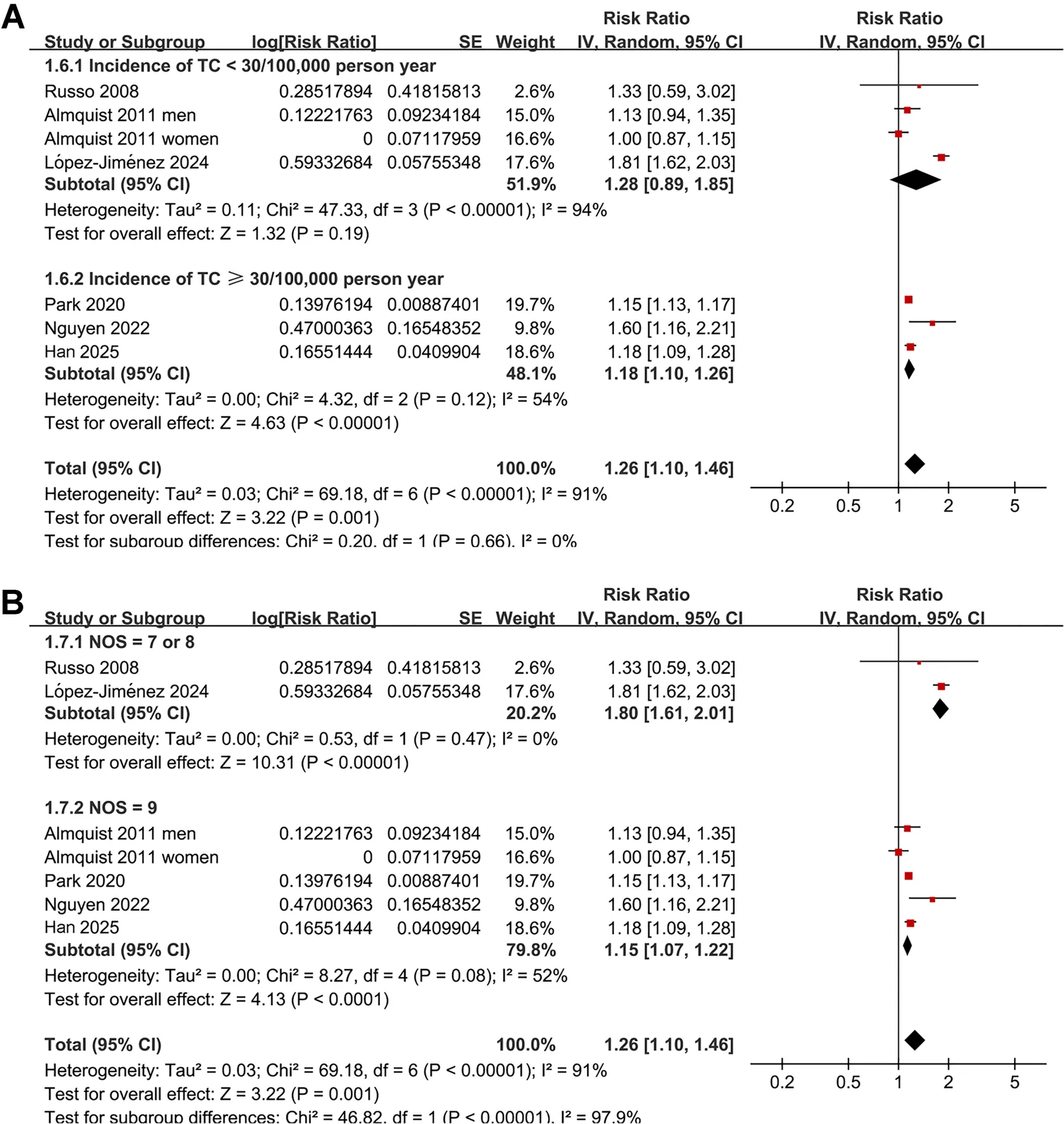
**Forest plots for subgroup analyses of the association between MetS and the risk of TC according to study-level TC incidence and study quality.** (A) Subgroup analysis stratified by the overall incidence of TC in each study, comparing cohorts with an incidence of <30 versus ≥30 per 100,000 person-years. The association between MetS and TC remained directionally consistent across incidence strata, and no significant subgroup difference was observed. (B) Subgroup analysis according to study quality, comparing studies with NOS scores of 7 or 8 versus those with a score of 9. Although the association remained statistically significant in both strata, the pooled effect estimate was stronger in studies with lower NOS scores, and the subgroup difference was significant. Squares represent study-specific effect estimates, with sizes proportional to study weight; horizontal lines indicate 95% CIs; and diamonds denote pooled subgroup and overall estimates generated using random-effects models. **Abbreviations:** CI, confidence interval; MetS, metabolic syndrome; NOS, Newcastle–Ottawa Scale; TC, thyroid cancer.

### Publication bias

As depicted in Figure 6, the funnel plots for the association between MetS and the risk of TC in adult populations appeared largely symmetrical. However, due to the small number of included datasets (*n* ═ 7), the ability to reliably detect publication bias was limited. Consistent with this, Egger’s test did not indicate statistically significant asymmetry (*P ═* 0.32), although this result should be interpreted with caution.

**Figure 6.**
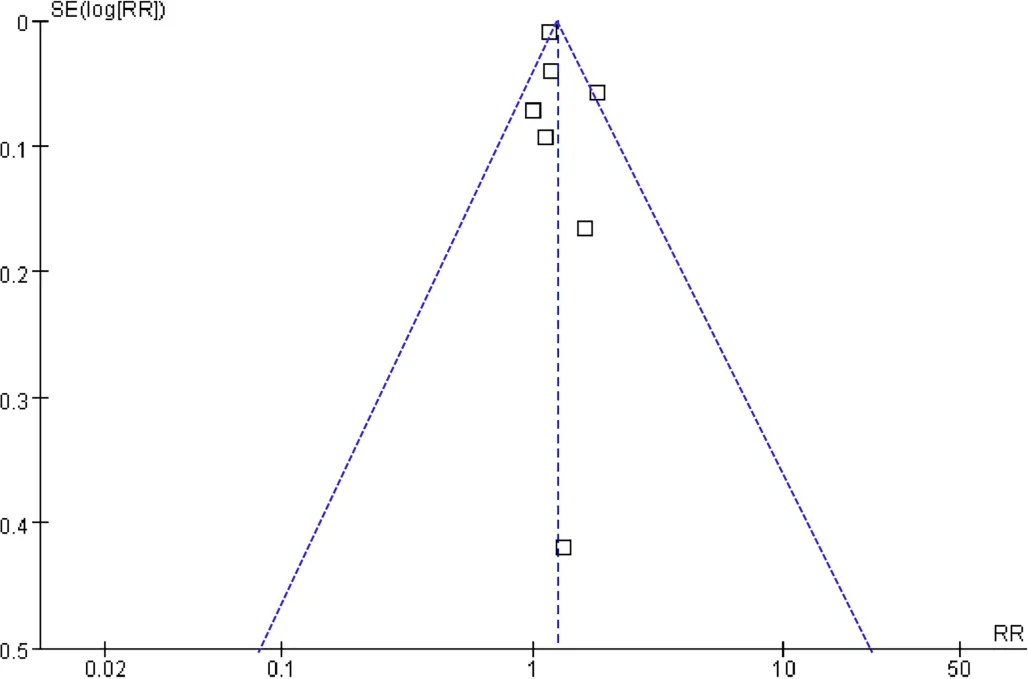
**Funnel plot assessing potential publication bias in the meta-analysis of the association between MetS and the risk of TC in adults.** Each square represents an individual dataset included in the meta-analysis. The x-axis shows the RR, and the y-axis shows SE(log[RR]). The vertical dashed line indicates the pooled effect estimate, whereas the oblique dashed lines represent the pseudo 95% confidence limits. Visual inspection suggests no marked asymmetry; however, interpretation should be cautious because the number of included datasets is small. **Abbreviations:** MetS, metabolic syndrome; RR, risk ratio; SE, standard error; TC, thyroid cancer.

## Discussion

This meta-analysis synthesizes updated longitudinal evidence indicating that MetS is associated with a modestly increased risk of developing TC in adults. Beyond demonstrating a statistically significant overall association, the findings extend earlier evidence where available data were sparse and largely inconclusive. Importantly, the observed relationship remained directionally consistent across multiple analyses, supporting the interpretation that clustered cardiometabolic dysfunction may serve as a marker of increased TC risk rather than establishing a causal relationship.

Several biological and clinical pathways may plausibly explain the higher TC risk conferred by MetS. Central obesity, a fundamental aspect of MetS, is linked to adipose tissue dysfunction and chronic low-grade inflammation. This condition is characterized by an increased secretion of pro-inflammatory cytokines, such as interleukin-6 (IL-6) and tumor necrosis factor alpha (TNF-α), elevated leptin levels, and decreased adiponectin. These factors contribute to a pro-proliferative and anti-apoptotic environment [26–28]. Insulin resistance and compensatory hyperinsulinemia are linked to the activation of insulin and insulin-like growth factor signaling pathways, which are implicated in thyroid cell growth and survival [29–31]. MetS is also associated with oxidative stress and immune dysregulation, which may contribute to DNA damage and tumor initiation [32]. However, these mechanisms remain hypothetical and do not establish causality in the context of the present findings. Additionally, hypertension and dyslipidemia may reflect endothelial dysfunction and altered lipid metabolism, further contributing to a tumor-promoting metabolic environment [33, 34]. Clinically, MetS frequently coexists with conditions such as nonalcoholic fatty liver disease [35] and obstructive sleep apnea [36], which may amplify systemic inflammation and hormonal disturbances relevant to thyroid tumorigenesis.

Interpretation of the subgroup analyses provides further insight into the robustness and context of the association. The direction of effect was broadly consistent across prospective and retrospective cohorts, across studies with varying sample sizes, and across strata defined by follow-up duration and baseline TC incidence, supporting the generalizability of the findings. Nevertheless, substantial heterogeneity was observed, suggesting that the magnitude of the association varies across populations and study settings. Differences in age structure, sex distribution, baseline metabolic risk profiles, healthcare utilization, and methods of TC ascertainment may partly account for this variability. Sensitivity analyses demonstrated that the overall association was not driven by any single study. Notably, studies with higher methodological quality (NOS = 9) demonstrated a more modest association compared with those of lower quality (NOS 7–8). This attenuation suggests that the strength of the observed association may be partly influenced by residual confounding or bias in studies with less comprehensive adjustment or methodological rigor. Accordingly, the findings should be interpreted as indicative of an association rather than a causal relationship.

Clinically, TC exhibits significant heterogeneity across sex, age, and histological subtypes, which may impact the observed association with MetS. In this analysis, sex-stratified subgroup results demonstrated broadly consistent associations in both men and women (Figure 4A), indicating that the relationship between MetS and TC risk is not confined to one sex. However, biological mechanisms may differ; sex hormones, particularly estrogen, have been implicated in thyroid carcinogenesis and may interact with metabolic abnormalities [37]. Additionally, the influence of MetS may vary across different age groups, potentially reflecting variations in hormonal status, metabolic profiles, and healthcare utilization, especially in postmenopausal women or older populations. Age-specific effects could not be robustly evaluated beyond study-level subgrouping. TC encompasses a range of heterogeneous histological subtypes, each with unique etiologies. These include follicular-derived cancers and syndromic forms, such as medullary thyroid carcinoma, which is linked to hereditary conditions like multiple endocrine neoplasia type 2 (MEN2) [3]. Because the included studies did not report results stratified by histological subtype, syndromic status, or detailed age- and sex-specific biological factors, these aspects could not be further explored, potentially representing additional sources of heterogeneity and residual confounding.

The results should be interpreted considering potential detection and screening biases. Cohorts may differ substantially in healthcare access, contact with medical services, and the intensity of diagnostic imaging, all of which can affect the likelihood of TC detection. Individuals with MetS are more likely to undergo routine clinical evaluations and imaging due to comorbid conditions, increasing the incidental detection of indolent or subclinical TC [38]. Consequently, variations in surveillance intensity across populations may inflate the observed incidence of TC independent of true biological risk. Although the included studies were longitudinal and adjusted for multiple covariates, differences in healthcare utilization and diagnostic practices could not be fully accounted for, potentially contributing to both the observed association and substantial between-study heterogeneity.

This meta-analysis has several strengths, including a comprehensive and up-to-date literature search, exclusive inclusion of cohort studies with longitudinal follow-up, and reliance on multivariable-adjusted estimates. Prespecified subgroup and sensitivity analyses further supported the robustness of the findings. However, several limitations warrant consideration. Some included studies were retrospective, which may introduce bias related to exposure misclassification or incomplete covariate adjustment [39]. Considerable heterogeneity existed across population sources, demographic characteristics, and methods used to diagnose TC, which could not be fully explored due to the lack of individual participant data. For instance, TC represents a heterogeneous group of malignancies with distinct histological subtypes that differ in pathogenesis and clinical behavior. However, the studies did not report results stratified by histological subtype, precluding subgroup analyses according to tumor type. This lack of granularity may have influenced the pooled risk estimate and should be regarded as a potential source of residual confounding. Furthermore, sensitivity analyses stratified by outcome ascertainment methods (e.g., cancer registries vs. administrative databases) or by comparable adjustment sets were not feasible, primarily due to the limited number of included studies and heterogeneity in reporting across studies. This limitation may contribute to residual heterogeneity and should be considered when interpreting the findings. Therefore, residual confounding due to differences in adjustment strategies across studies cannot be excluded and should be considered in the context of iodine intake, radiation exposure, family history, lifestyle behaviors, and medication use [40]. Additionally, surveillance and detection bias may have influenced the observed association, as individuals with MetS are more likely to undergo medical evaluations and imaging. Although TC outcomes were ascertained using generally reliable methods across studies—including cancer registries and claims databases often validated against registry data—some variability in outcome ascertainment exists. Given the limited number of included datasets, stratified sensitivity analyses according to outcome source were not feasible, and this potential source of heterogeneity should be acknowledged when interpreting the findings. Furthermore, although funnel plot inspection and Egger’s test did not indicate significant publication bias, these methods are inherently underpowered with a small number of included studies. Thus, the possibility of publication bias cannot be excluded and should be considered when interpreting the findings. Finally, while prespecified subgroup analyses were conducted to explore potential sources of heterogeneity, these analyses were based on a limited number of studies and median-based categorizations of study-level variables. Consequently, they should be regarded as exploratory and interpreted with caution, as the statistical power to detect true effect modification is limited, and the risk of chance findings cannot be dismissed. Therefore, the findings should be interpreted cautiously and do not support causal inferences.

From a clinical and public health perspective, these findings suggest that MetS may serve as a marker for increased TC risk, underscoring the broader relevance of metabolic health in cancer prevention. Given the modest effect size and the potential influence of detection bias, the results should be approached with caution and do not warrant changes in TC screening practices based solely on MetS status. Future studies should focus on well-designed prospective cohorts with standardized definitions of MetS, uniform TC outcome ascertainment, and careful adjustment for healthcare utilization. Research examining TC subtypes, tumor aggressiveness, and dose–response relationships across MetS components would further elucidate biological relevance.

## Conclusion

In conclusion, this meta-analysis indicates that MetS is associated with an increased risk of incident TC in adults. While the association appears consistent across multiple analyses, heterogeneity, residual confounding, and potential detection bias limit causal interpretation, highlighting the need for further high-quality research to clarify underlying mechanisms and clinical implications.

## Supplemental data

**Supplemental file 1.** Detailed search strategy for each database

**PubMed**

(“Metabolic Syndrome”[Mesh] OR “Insulin Resistance”[Mesh] OR metabolic syndrome*[tiab] OR insulin resistance syndrome*[tiab] OR syndrome X[tiab]) AND

(“Thyroid Neoplasms”[Mesh] OR thyroid[tiab]) AND (cancer*[tiab] OR neoplasm*[tiab] OR carcinoma*[tiab] OR malignan*[tiab] OR tumor*[tiab])

**Embase**

(‘metabolic syndrome’/exp OR ‘insulin resistance’/exp OR metabolic syndrome*:ti,ab OR insulin resistance syndrome*:ti,ab OR ‘syndrome x’:ti,ab) AND ( ‘thyroid tumor’/exp OR thyroid:ti,ab) AND (cancer*:ti,ab OR neoplasm*:ti,ab OR carcinoma*:ti,ab OR malignan*:ti,ab OR tumor*:ti,ab)

**Web of Science**

TS=((“metabolic syndrome” OR “insulin resistance syndrome” OR “syndrome X”) AND thyroid AND (cancer OR neoplasm OR carcinoma OR malignancy OR tumor OR malignant))

## Data Availability

All data generated or analyzed during this study are included in this published article.
